# The role of patient-derived ovarian cancer organoids in the study of PARP inhibitors sensitivity and resistance: from genomic analysis to functional testing

**DOI:** 10.1186/s13046-021-02139-7

**Published:** 2021-10-26

**Authors:** Mengyu Tao, Xia Wu

**Affiliations:** 1 Department of Gynecology and Obstetrics, Shanghai Key Laboratory of Gynecology Oncology, Renji Hospital, School of Medicine, Shanghai Jiao Tong University, 160 Pu Jian Road, Shanghai, 200127 People’s Republic of China; 2 Shanghai Key Laboratory of Gynecologic Oncology, Shanghai, 200127 People’s Republic of China

**Keywords:** PARP inhibitor, Patient-derived organoid, Ovarian cancer, Functional test, Predictive biomarker, Precise medicine

## Abstract

Epithelial ovarian cancer (EOC) harbors distinct genetic features such as homologous recombination repair (HRR) deficiency, and therefore may respond to poly ADP-ribose polymerase inhibitors (PARPi). Over the past few years, PARPi have been added to the standard of care for EOC patients in both front-line and recurrent settings. Next-generation sequencing (NGS) genomic analysis provides key information, allowing for the prediction of PARPi response in patients who are PARPi naïve. However, there are indeed some limitations in NGS analyses. A subset of patients can benefit from PARPi, despite the failed detection of the predictive biomarkers such as *BRCA1/2* mutations or HRR deficiency. Moreover, in the recurrent setting, the sequencing of initial tumor does not allow for the detection of reversions or secondary mutations restoring proficient HRR and thus leading to PARPi resistance. Therefore, it becomes crucial to better screen patients who will likely benefit from PARPi treatment, especially those with prior receipt of maintenance PARPi therapy. Recently, patient-derived organoids (PDOs) have been regarded as a reliable preclinical platform with clonal heterogeneity and genetic features of original tumors. PDOs are found feasible for functional testing and interrogation of biomarkers for predicting response to PARPi in EOC. Hence, we review the strengths and limitations of various predictive biomarkers and highlight the role of patient-derived ovarian cancer organoids as functional assays in the study of PARPi response. It was found that a combination of NGS and functional assays using PDOs could enhance the efficient screening of EOC patients suitable for PARPi, thus prolonging their survival time.

## Background

Epithelial ovarian, tubal, or primary peritoneal cancer (hereinafter referred to as EOC) represents the most devastating gynecologic cancer. Approximately 70% of EOC patients are at advanced stages at diagnosis [1, 2]. Despite initial therapy, normally consisting of surgical cytoreduction and platinum-taxane based chemotherapy, most patients with advanced-stage EOC will have a relapse of their disease and the five-year survival rate is below 45% [3, 4].

With great advances in the last decade in the understanding of the genetics of EOC, the introduction of poly ADP-ribose polymerase (PARP) inhibitors has significantly changed the approaches to EOC management. PARP inhibitors (PARPi) have been approved for the treatment of EOC in both relapsed and front-line settings due to their cytotoxic effects exploiting synthetic lethal, thereby killing tumor cells with homologous recombination deficiency (HRD) [5–7]. Approximately 41–50% of EOCs exhibit HRD involved in repair of DNA damage and replication [8, 9]. The best characterized causes of HRD in EOC are germline or somatic mutations in the *BRCA1* and *BRCA2* genes (*BRCA*) that encode the breast cancer type 1 and type 2 susceptibility proteins, which are detected in 12–15% and 5–7% of cases respectively [10–12]. The high prevalence of HRD is only partly explained by single gene mutations because, apart from *BRCA1/2*, only a very small number of other causal mutations in homologous recombination repair (HRR) related genes have been identified, such as *RAD51*, *ATM*, *ATR* and *PALB2* [8]. Clear evidence suggests that HRD can arise through germline and somatic mutations or methylation of a wider set of HRR related genes, or other as yet unidentified mechanisms [13]. Moreover, such mechanisms as reversion mutations in the *BRCA* genes can restore homologous recombination proficiency (HRP), revealing that HRD status is both a dynamic and complex phenotype [5].

HRD tests driven by next-generation sequencing (NGS) have been developed to better identify which cancers, except *BRCA* mutant, are eligible to receive PARPi. There are two main categories of HRD tests: (i) HRR pathway related genes that detect particular causes of HRD, (ii) mutational signatures or genomic ‘scars’ that calibrate the patterns of somatic mutations accumulated in HRD cancers irrespective of the underlying defects. These HRD tests provide key information to predict the PARPi response in patients who are PARPi naïve, however, these tests detect genomic scars that might not correlate well with PARPi sensitivity by current clinical assays [14–17]. A subset of patients can benefit from PARPi, despite the failed detection of the predictive biomarkers such as *BRCA1/2* mutations or HRD. Moreover, with the common use of PARPi for the management of EOC across the treatment life cycle, de novo and acquired resistance to PARPi have been found in patients [5]. Mechanisms of PARPi resistance are complex. Except for reversions or secondary mutations or other mechanisms to reinstate proficient HRR, those independent of HRR such as protection of replication forks can result in lack of PARPi response despite HRD [5, 18]. Given that there are multiple resistance mechanisms that may not be captured by the current HRD genomic assays, functional tests to assess current activity of HRR have the potential to provide a dynamic readout of actual, extant, HRR status. Therefore, combining genomic analysis with functional assays dissecting the specific DNA damage repair defects in a tumor might significantly enhance the selection of patients who are most likely to benefit from PARPi therapy. Recently, patient-derived organoids (PDOs) were reported to preserve the clonal heterogeneity and genetic features of original tumors and be likely a feasible and inexpensive model system for functional testing and interrogation of biomarkers [19–21].

In this review, we outline the mechanism of PARPi action, current predictive biomarkers for PARPi response, the available functional assays of HRD to asses PARPi sensitivity, and mechanisms of PARPi resistance. The limitations of genomic profiling in predicting response to PARPi are also addressed, and an important issue is well presented that functional assays dissecting the specific DNA damage repair defects in a tumor substantially improve the selection of candidates for PARPi. The benefits and significant achievement of PDOs in ovarian cancer research are then covered. PDOs are established as a promising tumor model that allows for rapid functional testing and prediction of therapeutic sensitivity to PARPi, where a combination of genomic analysis and functional testing of PDOs allows for the identification of targetable DNA damage repair defects, and also contributes to choosing rational combinatorial approaches for overcoming de novo or acquired resistance to PARPi.

### Mechanism of action of PARP inhibitors

DNA damage is a frequent event during cell life, which can be spontaneous or caused by environmental agents. It activates complex cellular process like DNA repair signaling and cell cycle regulation to maintain the integrity of the genome [22, 23]. Single strand breaks (SSB) are the most common DNA damage form, whereas double-strand breaks (DSB) are most cytotoxic [24]. Proteins of PARP family play a critical role in SSB repair [25]. PARP1, that accounts for up to 90% of the entire PARP activity, detects disruptions in replication forks and binds damaged DNA at SSB, causing a series of allosteric changes in the structure of PARP1 and the closely related PARP2 protein to activate their catalytic function [26]. This leads to the PARylation and recruitment of DNA repair effectors as well as the remodeling of chromatin structure around damaged DNA [27, 28]. After completing this recruitment role, PARP auto-PARylation triggers the release of bound PARP from DNA to allow other DNA repair components to work [29, 30]. Homologous recombination repair (HRR), generally a “conservative” mechanism, repairs DNA DSB in a high-fidelity way by using the homologous DNA sequence when available, and the activities of key molecules including BRCA1/2 and RAD51 among others [31–33]. If an undamaged template DNA is unavailable, the fast but error prone Non-Homologous End Joining (NHEJ) repair pathway is the primary method of DNA DSB repair utilizing essentially a direct ligation approach [5, 32]. When HRR pathway is altered, leading to HRD, non-conservative forms of DNA repair predominate with a less preserved genomic integrity, which may foster cancer initiation or progression [31, 32]. Thus, tumor cells with HRD show a greater reliance on PARP activity for survival maintenance [5].

In 2005, the finding that single-agent PARP inhibition selectively killed BRCA1or BRCA2 deficient cells was a pivotal discovery that ushered in clinical oncology [34, 35]. This was soon followed by the demonstration that non-BRCA deficiencies in the HRR pathway also resulted in PARPi sensitivity [36, 37]. The original rational mechanism underlying synthetic lethal was that PARP inhibition in HRD tumor cells caused persistent SSB, thereby inducing the collapse of replication folks and the formation of DSB, ultimately resulting in chromosome deletions, translocations, and subsequent cell death [34, 35]. Conversely, normal cells with functional HRR are able to deal with the DSB accurately and effectively. Additionally, Murai et al. demonstrated that PARP trapping is strongly related to PARPi cytotoxic potency [7]. This activity stabilizes PARP1 and PARP2 DNA complexes preventing auto-PARylation and PARP1 release from the site of damage and therefore interfering with the catalytic cycle of PARP1 [7, 38]. Despite similar activity in PARP catalytic inhibition, PARPi have differing effects in terms of their ability to “trap” PARP on DNA. Talazoparib shows the strongest in vitro cytotoxicity and PARP trapping [38], followed by Niraparib. Olaparib and Rucaparib show a medium cytotoxicity and PARP trapping, whereas Veliparib is the weakest one [39]. These differences in PARP1 trapping and cytotoxicity need to be taken into account when designing combination therapies.

### Predictive biomarkers of PARP inhibitors response

**Fig. 1:**
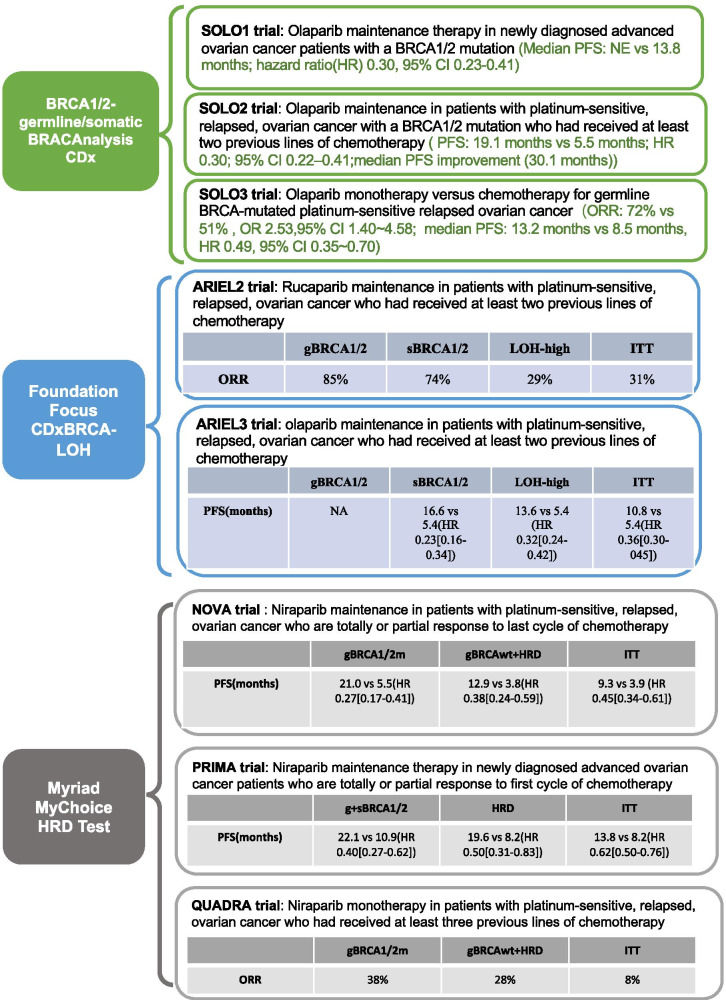
Clinical studies investigating the predictive value of HRD in ovarian cancer measured by commercial genomic sequencing. Abbreviations: PFS = progressed free survival; NE = not end; HR = harzd ratio; gBRCA1/2 m = germline BRCA1/2 mutation; LOH = loss of heterozygosity; ORR = overall response rate; ITT = intention to treat; gBRCAwt = germline BRCA wildtype; HRD = homologous recombination deficient

PARPi as maintenance therapy have been confirmed beneficial in terms of progression free survival (PFS) or overall survival (OS) in platinum sensitive recurrent ovarian cancer by a series of clinical trials, especially for those with *BRCA1*/2 mutations [14–17]. There is a clear association between platinum sensitivity, defined based on the time gap from last platinum exposure to disease progression, and response to PARPi [40]. Hence, clinical trials in ovarian cancer shift towards using PARPi as maintenance therapy in patients who respond to platinum-based chemotherapy (Table 1). With the release of data exploring the role of PARPi as first-line maintenance therapy, in December of 2018, the US FDA expanded the use of olaparib as frontline maintenance therapy in patients with ovarian cancer (see also Table 1). Compared with chemotherapeutics, PARPi are less toxic as far as they allow to maintain the quality of life during palliative treatment. A variety of PARPi have been approved by the US FDA for the late-line monotherapy in recurrent ovarian cancer with *BRCA* mutations or those who are both HRD-positive and platinum-sensitive, providing new treatment options for such patients (see also Table 1). HRD status plays an important role in identifying eligible patients for PARPi. The HRD tests that are used in the clinic or have been tested within published randomized clinical trials to date measure a genotype (gene mutation/methylation or genomic scar) that correlates with an HRD phenotype and deficient HRR (Fig. 1).

**Table 1:** Approved indications for PARPi by FDA & NMPA (in order of approval time)

Drug	Approved Time	Application	Indication	Clinical Trials
Olaparib	2014.12 (FDA)	Late-line monotherapy	Adult patients with advanced recurrent HGSOCs with germline BRCA mutations, who have received three or more prior lines of chemotherapy	Study 42
Rucaparib	2016.12 (FDA)	Late-line monotherapy	Adult patients with advanced recurrent EOCs with germline BRCA mutations, who have received two or more prior lines of chemotherapy	Study 10 & ARIEL2
Niraparib	2017.3 (FDA)	Maintenance therapy	Adult patients with recurrent EOCs, who had either a complete or a partial response to platinum-based chemotherapy	NOVA
Olaparib	2017.8 (FDA)	Maintenance therapy	Adult patients with recurrent EOCs, who had either a complete or a partial response to platinum-based chemotherapy	Study 19 & SOLO-2
Rucaparib	2018.4 (FDA)	Maintenance therapy		ARIEL3
Olaparib	2018.8 (NMPA)	Maintenance therapy		Study 19 & SOLO-2
Olaparib	2018.12 (FDA)	Maintenance therapy	Newly diagnosed adult EOC patients with BRCA mutations, who had either a complete or a partial response to first-line platinum-based chemotherapy (frontline maintenance therapy)	SOLO-1
Niraparib	2019.10 (FDA)	Late-line monotherapy	Recurrent adult EOC after three or more prior lines of chemotherapy: who are BRCA mutated or HRD-positive and platinum sensitive	QUADRA
Olaparib	2019.12 (NMPA)	Maintenance therapy	Newly diagnosed adult EOC patients with BRCA mutations, who had either a complete or a partial response to first-line platinum-based chemotherapy (frontline maintenance therapy)	SOLO-1
Niraparib	2019.12 (NMPA)	Maintenance therapy	Adult patients with recurrent EOCs, who had either a complete or a partial response to platinum-based chemotherapy	NOVA

#### HRR pathway related gene panels

Testing for germline and somatic mutations in *BRCA1/2* and other HRR-related genes may be used to infer the presence of HRD. Clinical studies have demonstrated that mutations in other HRR genes have a similar positive impact on overall survival and platinum responsiveness as *BRCA1/2* mutations [41, 42]. In a retrospective analysis from Study 19, twentyone high-grade serous ovarian cancer (HGSOC) patients without *BRCA* mutations had at least one loss-of-function mutation in candidate HRR genes including *BRIP1*, *CDK12*, *RAD54L*, *RAD51B*, *ATM*, *FANCA*, *FANCD2*, *FANCL*, *RAD51C*, *RAD52* and *XRCC3* [43]. The cohort of HGSOC that lacked a *BRCA* mutation but carried a mutation in other HRR genes derived a similar benefit to those with a *BRCA* mutation (HR 0.21 and HR 0.18, respectively) and this was of a greater magnitude than that observed in the cohort that lacked mutations in either *BRCA* or HRR genes (HR 0.71) [43]. Nevertheless, due to the relative rarity of non-*BRCA* HRR gene mutations, these studies grouped together all HRR genes except *BRCA,* which makes it difficult to interpret the relevance of any individual HRR gene at present. Though mutations or methylation of *RAD51* and *ATM* alterations were identified associated with PARPi responses in ovarian cancer patients [44, 45], the extent to which other candidate HRR related genes impact PARPi response is still under investigation. Mutations in non-*BRCA* HRR genes are not currently part of an FDA-approved test to assess PARPi eligibility in ovarian cancer. Nonetheless, some clinicians identify patients who may benefit from PARPi based on mutations in HRR pathway genes.

A major challenge of *BRCA* and wider HRR gene testing is determining the clinical relevance of variants of uncertain significance (VUS) that are typically rarer missense mutations and also include intronic or exonic mutations that may alter RNA splicing [46–48]. The problem of VUS is more pronounced for wider gene panel tests where the functional and clinical consequences of most individual genomic loci are not well characterized, and individual mutations are not highly recurrent. Furthermore, somatic VUS may be more numerous and diverse than germline variants as they may arise in the context of an elevated mutation rate and/or genomic instability [49]. Thus, many variants remained of uncertain (or unknown) significance cannot be included, which could affect the accuracy of predicting HRD status and PARPi responses based upon alterations in HRR genes. In addition, due to intrinsic technological limitations of NGS including limited sensitivity compared with digital polymerase chain reaction (PCR), the analytic validity of epigenetic alterations that could be involved in HRD such as *BRCA1* or *RAD51C* promoter methylation seems not to be sufficiently reliable. Given the difficulty of predicting the functional relevance of an individual point mutation or structural variant within a given gene footprint, corroborating evidence of HRD from a genomic mutation/scar test and/or a functional assay, as discussed below, would ideally be acquired.

#### Genomic signatures and scars

“Genome scars” consist of specific patterns of mutations and structural aberrations of chromosomes, including rearrangements, gains, and losses of DNA. Quantitative measures of HRD-associated chromosomal abnormalities have been developed based on studies of *BRCA1/2* deficiency and could assess up to three types of genomic scarring patterns: loss of heterozygosity (LOH), telomeric allelic imbalance (TAI), and large-scale transitions (LST) [50–52]. Measuring these genomic features helps to identify cancers with a history of HRD, irrespective of the underlying aetiology. The most common genomic scar assays reported to date are two commercially available tests that combine *BRCA* mutation testing with a genomic instability score derived from the unweighted sum of TAI, LST and LOH (myChoice HRD test, Myriad Genetics) or with an assessment of fraction of genomic sub-chromosomal LOH (FoundationFocus CDxBRCA, Foundation Medicine) [53, 54]. HRD tests provide key information to predict the PARPi response in patients who are PARPi naïve. However, genomic scars are permanent despite dynamic changes in HRR function, and HRD testing via one of these assays on archival tumor may not represent the current HRD status of the cancer cells, therefore, the test results might not always correlate well with PARPi sensitivity in the current state. A subset of patients can actually benefit from PARPi, despite the failed detection of the predictive biomarkers such as *BRCA1/2* mutations or HRD. ARIEL3, a randomised, double-blind, placebo-controlled, phase 3 trial, assessed rucaparib versus placebo as maintenance treatment after response to second-line or later platinum-based chemotherapy in patients with high-grade, recurrent, platinum sensitive ovarian carcinoma. The NGS assay combines mutation analysis of *BRCA1/2* with measurement of the percentage of genome-wide LOH in the cancer tissue (FoundationFocus CDxBRCA) as a biomarker for sensitivity to rucaparib treatment. The result shows that the non-*BRCA* mutated LOH-low group also benefited significantly from rucaparib compared to placebo (Fig. 1), suggesting that the discriminating power of the LOH testing for selecting HRD tumors in ovarian cancer is suboptimal [16, 44]. The phase III NOVA trial, which prospectively assessed the MyChoice assay in the maintenance setting following platinum-based chemotherapy, aimed to broaden the efficacy population to those who are HRD-positive without *BRCA* mutation [15]. Findings echoed those of ARIEL3, including an intermediate benefit in the *BRCAwt*/HRD and failure to identify an HRP group who do not benefit (Fig. 1) [15]. According to clinical trials assessing the predictive value of several approaches using NGS based mutational signatures in PARPi naïve EOC patients, they have been revealed to produce both false-positive and false-negative results with regard to the prediction of PARPi sensitivity, which strongly indicates the need for complementary functional assays.

In addition, with the common use of PARPi for the management of EOC across the treatment life cycle, an increasing number of patients have been exposed to prior PARPi with/or without progression on PARPi, hence, the efficacy of PARPi after PARPi treatment need to be explored. Unfortunately, many clinical trials excluded patients who had prior exposure to PARPi, as such, data regarding the efficacy of repeated use of PARPi for recurrent disease of patients who applied PARPi as first-line maintenance treatment remain limited. De novo and acquired resistance to PARPi with various mechanisms have been found in patients, and PARPi resistance mechanisms independent of HRR can result in lack of PARPi response despite HRD (it will be discussed in the next section). Identification of biomarkers for patients who would benefit from PARPi retreatment is currently underway. A prospective randomized controlled trial OReO/ENGOT Ov-38 that sought to assess the efficiency of olaparib retreatment in platinum sensitive recurrent ovarian cancer started in June 2017 (NCT03106987). According to data presented at the 2021 European Society for Medical Oncology Congress, results of OReO/ENGOT Ov-38 trial showed that rechallenge with maintenance olaparib after platinum-based chemotherapy response resulted in longer PFS for patients with relapsed ovarian cancer, regardless of their BRCA status.

#### Functional assays to asses PARPi sensitivity

To date, all of the genomic assays available for HRD have some limitations. Those to detect genomic scars provide snapshots in time measuring past events, but may not reflect the current state. In order to identify real-time HRD, functional assays may produce better evaluation of the dynamic readout of actual, extant, HRR status of the tumor. The complexity of measuring all proteins of interest within a pathway makes it very desirable for a functional assay of HRD to measure a single downstream event integrating multiple upstream components of the HRR pathway. The most common functional assays involve quantification of RAD51 foci. In the process of HRR, RAD51 protein is loaded onto the ends of DSBs, allowing the resected DSBs to invade the sister chromatid. RAD51 forms distinct subnuclear foci after DNA damage, and the inability to form RAD51 foci is a common feature of HRD. This is a functional read-out for HRD without defining the actual cause of HRD. It is the presence of the RAD51 foci that identifies the functional status for homologous recombination up to the stage for RAD51 loading [55]. Since HRR is a dynamic process and relates to cell cycle, false positive HRD results can arise when tumor cells are senescent, as, owing to cell cycle arrest, they have absent RAD51 nuclear foci irrespective of BRCA status.

At first, RAD51 foci were determined by immunochemistry (IHC) staining on fixed breast cancer biopsies collected before and 24 h after neoadjuvant treatment with DSB-inducing chemotherapy [56, 57], and geminin was utilized as the marker of S/G2 phase of the cell cycle [57]. The absence of chemotherapy induced RAD51 foci in geminin positive tumor cells was taken as a sign of HRD. A significant inverse correlation was found between the RAD51 score and the tumor response rate after chemotherapy [56, 57]. RAD51 foci were assessed in a retrospective study evaluating the HRD status of samples collected from EOC patients before and after chemotherapy. The decrease in the number of RAD51 foci on IHC stained samples did not predict an improved prognosis, but a change from high to low RAD51 detection on following chemotherapy was associated with good prognosis [58]. However, this approach may overestimate HRP, as deficient DNA repair can restore a low level of residual foci in 24 h following chemotherapy.

In general, methods for assessing the accumulation of HRR pathway protein in treated biopsies tend to retrospectively evaluate drug response but lack predictive value. To avoid ineffective drug exposure, an improvement of functional analysis is the induction of DSBs before starting treatment of patients. Most commonly, fresh tumor tissue is collected and DSBs are induced ex vivo by ionizing radiation (IR) [59, 60]. The homologous recombination REpair CAPacity (RECAP) test developed by Meijer et al. was first performed to assess the formation of RAD51 foci in proliferating cells (in S/G2-phase) following irradiation of fresh breast cancer samples as well as fresh primary ovarian cancer cells [61]. Fresh biopsies were irradiated ex vivo and subsequently cultured for 2 h before RAD51 foci were visualized, and then formalin-fixed and paraffin-embedded (FFPE). Immunofluorescence (IF) was then used to detect RAD51 foci and geminin expression. A lower percentage of proliferating cells with RAD51 foci was demonstrated to be associated with PARPi-sensitive models and a higher percentage associated with PARPi-resistant models. This RECAP assay was also applied in tissue slices and ascites-derived cancer cells from ovarian tumors but cell cycle controls were missing [62].

Despite the potential advantages of RECAP assay, its introduction into routine practice is limited by the need for fresh tumor specimens, ex vivo DNA damage by irradiation, and the need for proliferating cells. To address this, an approach which relies on the presence of endogenous DNA DSB in the tumor at the moment of biopsy collection was studied in two small studies [63, 64]. RAD51 nuclear foci were detected in treatment naive, FFPE tumor samples, and it was shown that low levels of RAD51 foci in untreated tissue samples associate with clinical response to PARPi of breast cancer patients [63, 64]. These results suggest that reduced RAD51 foci in untreated tumor tissue may be a surrogate of HRD, indicating that clinical application of this assay might be possible. Notably, retrospective analysis of RAD51 foci in the TOPACIO trial did not show statistically significant association with response to niraparib plus pembrolizumab in a platinum-resistant ovarian cancer cohort [65]. Overall, there are such challenges to the RAD51 focus formation assay as source of tissue, timing of RAD51 focus assessment, mode of evaluation of RAD51 foci, modality of DNA damage used, issues with microscope resolution, and standardizing the definition of RAD51 positive cells. Additional correlation with treatment response is also needed. Robust and well controlled studies to evaluate and improve the RAD51/geminin score evaluation system also need to be carried out in ovarian cancer.

Using ex vivo, in vivo or in vitro approaches, patient-derived three-dimensional (3D) models are now used in the field of oncology widely in functional assays to evaluate PARPi sensitivity and identify new biomarkers so as to predict patient clinical outcome. 3D tumor models using freshly resected samples, such as solid tumors (localized or metastatic), spheroids (from fluid paracentesis) or circulating tumor cells (from blood) are used to assess the therapeutic effects of PARPi. It was shown in some studies that short-term cultured PDOs can directly or indirectly predict the PARPi sensitivity of individuals through drug susceptibility tests and DNA damage repair functional assays. Moreover, for PARPi resistant patients, drug screening by PDOs may guide the initial therapy or combination therapy precisely.

### Mechanism of PARPi resistance and combination therapy strategies

**Fig. 2:**
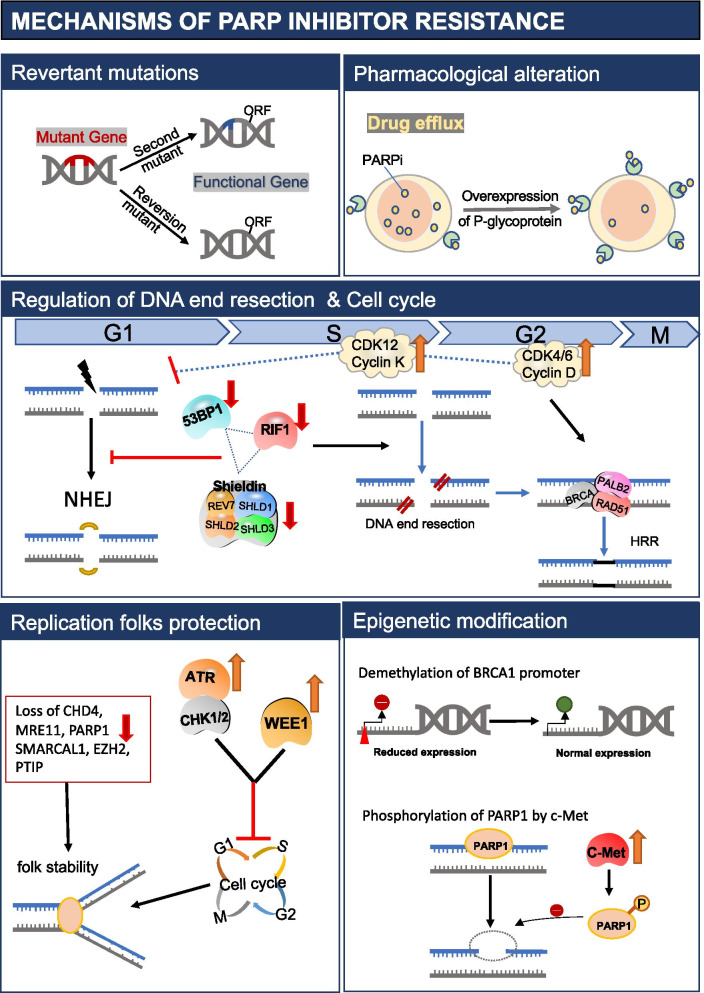
Acquired resistance to PARPi can develop via three general mechanisms: restoration of HRR owing to (i) revertant mutations, (ii) demethylation of BRCA1 promoter or (iii) regulation of DNA end resection and cell cycle; replication fork protection; and drug target-related effects, such as (i) upregulation of drug efflux pumps or (ii) phosphorylation of PARP1

The incorporation of PARPi as standard first-line maintenance treatment transformed the identification of patients with inherent or acquired PARP resistance into a new urgent need. Numerous mechanisms of PARPi resistance have been described in pre-clinical and clinical studies (Fig. 2). Acquired resistance to PARPi can develop via three general mechanisms: restoration of HRR owing to restoration of BRCA1/2 function or loss of DNA end-protection; restoration of replication fork stability; or drug target-related effects, such as the upregulation of drug efflux pumps or mutations in PARP or functionally related proteins.

Secondary “revertant” mutations in *BRCA1* or *BRCA2* that restore the open reading frame of the genes and sufficient HRR function have been fully validated as a mechanism of resistance to PARPi, which also causes platinum-based chemotherapy resistance [66–69]. Secondary mutations in *BRCA1/2*, *RAD51C*, *PALB* and other HRR-related genes that restored the open reading frame (ORF) was identified in paired post-progression biopsies [44, 70]. Such reversions may be detected in a fresh tissue sample that is resistant to therapy, while testing on germline or archived tumor specimens may not reflect the reversion mutation and return of HRR function. In addition, there are other mechanisms of treatment resistance that would not be captured by the DNA sequencing.

DNA end resection is the key of DNA repair choices and often stimulated in the S/G2 phase of cell cycle to promote HRR [71]. In the G1 phase, 53BP1 localizes to DSB sites and interacts with RIF1, to block the recruitment of BRCA1 [72, 73]. Shieldin, an effector complex composed of SHLD and REV7, is recruited by 53BP1 to protect DNA ends from resection [74, 75]. It is demonstrated that loss of function of these proteins leads to the process of DNA end resection and contributes a lot to HRR restoration and PARPi resistance [76–78]. DNA end resection also depends on cyclin-dependent kinase (CDK) activity, thus loss of CDK12 function could disrupt HRR and sensitize ovarian cancer cells to PARPis [79].

In addition to DNA repair, PARP1 and BRCA1/2 participate in DNA replication. PARP1 plays a key role in mediating the accumulation of regressed forks and avoiding DSB formation [80]. BRCA1/2 protect nascent DNA at stalled replication forks from degradation [81, 82]. When PARPi trap PARP1 on DNA to block DNA replication, cells will rely on BRCA1/2 to protect replication forks. As BRCA is defective, the absence of DNA replication folk protection leads to genome instability and cell death. Recently, it was suggested that DNA replication fork protection but not HRR caused PARPi resistance in *BRCA*mut cells and patients, which challenged the HRR dominance in synthetic lethality [83]. As more and more research focused their attention on mechanisms of fork protection, many mechanisms leading to resistance to PARPi have been revealed [84]. MRE11 and MUS81 are nucleases whose activity is required for the processing of stalled replication forks. In the absence of BRCA1/2, uncontrolled resection of unprotected stalled forks by MRE11 leads to fork collapse and contributes to increased genomic instability [85]. In line with this observation, depletion of the MLL3/4 complex protein PTIP or the nucleosome remodelling factor CHD4 prevents MRE11 recruitment to stalled forks, resulting in fork protection and resistance to PARPi in BRCA1/2-deficient cells [86, 87]. The chromatin remodelling complex SMARCAL1 has also been shown to promote the MRE11-dependent degradation of nascent DNA in BRCA1/2-deficient cells. In a manner like loss of PTIP, SMARCAL1 depletion decreases the sensitivity of BRCA1-deficient tumour cells to PARPi although this effect seems to be cell-type specific [88]. SLFN11 is another factor implicated in replication stress. Upon replicative damage, cells undergo irreversible cell-cycle arrest at the G1/S phase, mediated by the engagement of SLFN11 with the replication helicase complexes. Consequently, loss of SLFN11 enables cells to progress through the S phase in the presence of replicative stress thereby decreasing the cytotoxicity of PARPi [89]. Limiting the recruitment of MUS81 through inhibition of the methyltransferase EZH2 has also been shown to result in fork protection and partial resistance to PARPi [90]. Finally, PARP1 is known to mediate the recruitment of MRE11 to stalled replication forks [26]. Downregulation of PARP1 before BRCA1/2 loss restores the stability of stalled forks and promotes cell survival, likely by limiting the accumulation of MRE11 at replication forks [91].

Epigenetic modification may affect PARPi sensitivity and lead to resistance. Multiple lines of treatment prior PARPi lead to loss of *BRCA1* promoter methylation, which rescues the expression of BRCA1 and confers resistance of PARPi [92]. Phosphorylation of PARP1 at Tyr907, mediated by c-Met, increases PARP1 enzymatic activity and reduces its binding to PARPi, thereby rendering cancer cells resistant to PARPi [93]. Pharmacological alteration also modulates PARPi response. As some of PARPi are substrates of multidrug resistance protein MDR1(P-glycoprotein) encoded by ABCB1 gene, studies indicate that the enhanced P-glycoprotein mediated drug efflux contributes to the acquired resistance to PARPi [94].

Further studies are required to develop therapeutic strategies that combat or delay the emergence of acquired resistance. The rationale for the exploit of efficacious PARPi combinations has generally focused on enhancing the antitumor effect of PARPi by creating DNA damage or modulating DNA repair. The strategy of combining PARPi with targeted agents like WEE1, ATR and CHK1/2 inhibitors that impair the ability of tumor cells to stall the cell cycle to process and repair “trapped” PARP-DNA complex has gained some attention. Multiple inhibitors of cell-cycle checkpoint kinases are being developed by pharmaceutical companies and are being tested in clinical trials designed to assess anticancer efficacy in combination with PARPi (e.g., NCT02264678, NCT02576444). In addition, pre-clinical evidences suggest that a series of targeted agents, such as inhibitors of the phosphatidylinositol 3-kinase (PI3K), CDK and histone deacetylases (HDAC) can induce or enhance a BRCAness phenotype in tumor cells, thus causing sensitivity and overcoming resistance to PARPi [95–97]. Data from early phase demonstrate an efficacy (ORR 36%) of olaparib and the α specific PI3K inhibitor apelisib, which provides preliminary clinical evidence of synergism between olaparib and alpelisib, particularly in EOC [98]. Furthermore, PARPi could also be effectively combined with drugs that target specific features unrelated to the DDR function. The proof-of-concept EVOLVE study (NCT02681237) assessed cediranib-olaparib combination therapy after progression on a PARPi [99]. Interestingly, the activity of cediranib-olaparib varied according to the PARPi resistance mechanism, and patients with reversion mutations in homologous recombination genes and/or ABCB1 upregulation had poor outcomes.

Resistance to PARPi might be an inevitable consequence of the genomic instability of the HRD tumors. Although targeted approaches designed to overcome resistance to PARPi remain limited, the systematic identification of the vulnerabilities of PARPi resistant tumors will therefore be an important step. An improved understanding of the biology of PARPi resistant tumors will facilitate the development of rational combination treatment strategies to prevent and/or delay the onset of resistance and will ultimately lead to improved long-term outcomes for patients. Although a subset of genetic reversion events leading to resistance to PARPi in patients with *BRCA*-mutated cancers can be readily detected by NGS, other mechanisms of resistance to PARPi, such as restoration of HRR or replication fork protection, might be more challenging because these processes are often caused by genomic alterations, such as large deletions or breakpoints in introns, which are less likely to be detected using current sequencing approaches. Thus, there is a critical need for the development of HRD functional assays, which could, as a complementary to genomic studies, predict tumor response in real-time, identify PARPi resistance mechanisms and adopt alternative therapeutic strategies.

### Patient-derived ovarian cancer organoids overview

One of the major hurdles for the development of novel regimens for cancer treatment is the challenge of translating results from bench to bedside, which is mainly due to that many cancer models poorly recapitulate patient’s tumor, and as a consequence, many drugs that perform well on preclinical cancer models ultimately fail in clinical trials. Although cancer cell lines and patient-derived xenografts (PDXs), as commonly used human cancer models, contributed a lot to cancer research, they have a number of drawbacks. For instance, success rate of establishing cell lines is generally low and involves extensive adaptation and selection to in vitro two-dimensional (2D) culture conditions, thus, the resulting cell lines are the selection of some specific clones [100]. PDXs are generated by transplanting freshly derived patient material subcutaneously or orthotopically into immunodeficient mice, which is commonly applied in preclinical testing of novel therapies for cancer treatment. Compared to cell lines, PDXs biologically mimic the primary tumor and reliably recapitulate the tumor microenvironment, such as the 3D structure, the interaction of cancer cells with stroma and blood vessel infiltration and even the immune responses. Nevertheless, xenografts, involving limited engraftment efficiencies and significant investments in resources for their maintenance, are poorly suited for high-throughput drug screening or for genetic manipulation. Moreover, the approach is time consuming and may undergo mouse-specific tumor evolution [101]. To overcome these drawbacks, organoid as a potential promising preclinical platform has been an emerging field in the past decade.

Organoids are 3D in vitro culture systems derived from self-organizing stem cells. The ability to grow organoids with high efficiency from healthy human adult stem cells has paved the way to grow organoids from patient-derived tumor tissue. Briefly, the culture of PDOs begins with mincing up the patient tissue and embedding the cells into Matrigel or Basement Membrane Extract, which are two kinds of solubilized basement membrane extracts derived from Engelbreth-Holm Swarm mouse sarcoma consisting of laminin, collagen IV, entactin, and heparan sulfate proteoglycans. Then, cells are cultured under serum-free conditions and grow into spheroid shapes that are termed “organoids” [102]. PDOs, generated from different sources such as primary and metastatic tumors, blood tissue, ascites, pleural effusion drainage, and organized into 3D structures, retain the genetic landscape and histological properties of the original tumor. To date, long-term or short-term patient-derived organoid cultures have been successfully established from different types of cancers like liver [103], prostate [104], breast [105], colon [106], gastric [107], ovarian cancers [108], and others.

In 2015, Kessler M et al. demonstrated the establishment of long-term, stable 3D organoid cultures from human fallopian tubes which also respond to estradiol and progesterone treatment in a physiological manner [109]. This study essentially revealed the existence of fallopian tube stem cells. The first hallmark study established 33 short-term cultured (within 3 passages) organoid lines generated from ascitic or pleural fluid of 22 HGSOC patients and 1 LGSOC patient with nearly 100% success rate [110]. Intriguingly, high organoid formation rates were reported even from neoadjuvant treated patients provided the tumor was macroscopically visible. These PDOs have well preserved the original tumor histological and genetic characteristics and can be used for drug sensitivity testing and DNA repair functional assays [110].

Kopper et al. reported for the first time the long-term cultured (over 30 passages) patient-derived ovarian cancer organoids with derivation efficiency of 65% and established a biological sample library of 56 organoid lines from 32 ovarian cancer cases which covers all main histological subtypes [108]. Moreover, organoids established from normal fallopian tube and OSE, which are obtained from *BRCA* germline mutation carriers undergoing prophylactic bilateral salpingoophorectomy, were subjected to p53 inactivation to model HGSOC. What is noteworthy is that, even after over ten times of passage, PDOs still retain histological and genomic features of the pertinent lesion from original tumors, illustrating intra- and inter-patient heterogeneity, and can be genetically modified. It revealed that PDOs can be used for drug-screening assays and capture different tumor subtype responses to the gold standard platinum-based chemotherapy, including acquisition of chemoresistance in recurrent disease. Furthermore, EOC organoids can be xenografted, enabling in vivo drug-sensitivity assays. This is the first large-scale study presenting a long-term cultured PDO platform that enables in vitro expansion, manipulation and analysis of a wide variety of EOC subtype, indicating a major step in EOC research.

Recently, several researches with smaller samples have also demonstrated the successful establishment and application of ovarian cancer PDOs, which maintain good consistency with the original materials, and are feasible for drug sensitivity testing [111–116]. Hoffmann et al. established 15 organoid lines from HGSOC primary tumor deposits that closely match the mutational profile and phenotype of the parental tumor. They found that Wnt pathway activation leads to growth arrest of these cancer organoids and active BMP signaling is almost always required for the generation of HGSOC organoids, while healthy fallopian tube organoids depend on BMP suppression by Noggin [116]. Maenhoudt et al. tested multiple culture medium components, identified neuregulin-1 as a key factor in maximizing EOC organoid development and established expandable PDOs from HGSOCs with a derived efficiency of nearly 40% [111]. Maru et al. modified a Matrigel bilayer organoid culture protocol to cope with the digestion-resistant nature of EOC and established a total of nine propagated PDOs [112]. Chen et al. developed short duration organoid cultures of multicellular spheroids (MCSs) from ovarian cancer malignant effusions and used them as a platform for empirical drug sensitivity testing [113]. In addition, there are also some preclinical studies that utilized PDOs as a disease model to verify the newly discovered molecular mechanisms and targeting strategies in different subtypes of ovarian cancer [117–122].

Collectively, PDOs provide benefits like high derivation efficiency, 3D spheroid structure, tumor heterogeneity preservation, matched normal controls and are compatible with high throughput drug screening. Thus, the organoid platform might be potentially promising in drug discovery and personalized medicine. Three trials are currently ongoing to evaluate the role of PDOs in predicting the clinical efficacy of anti-cancer drugs (chemotherapy and targeted therapy) in EOC (NCT04279509, NCT04768270 and NCT04555473). The NCT04279509 trial is a single-center study aimed at prospectively determining if high-throughput drug screen assays using PDOs can accurately select chemotherapeutic agents that result in objective response in patients with refractory solid tumors (head and neck squamous cell carcinoma, colorectal, breast cancer and EOC). NCT04768270 is a single-center study aimed at verifying whether PDOs can help guide precision treatments for EOC patients. NCT04555473 is a longitudinal observational phase II study of the reliability of HGSOC PDOs as a model for the patients’ response to treatments.

### PDOs in study of PARP inhibitor sensitivity and resistance

Cancer drug screening in patient-derived cells holds great promise for personalized oncology and drug discovery but lacks standardization. Recent research demonstrated that PDOs could likely be a promising platform for rapid assessment of susceptibility to clinical drugs including PARPi. Using DeathPro, an automated microscopy-based assay assessing cell death and proliferation inhibition, Julia et al. operated a cell sensitivity test in monolayer or organoid culture from ovarian cancer patients with clinically relevant drugs [123]. Interestingly, drug effects in organoids were more diverse and had lower therapeutic potential. Genomic analysis revealed links between drug sensitivity and DNA repair deficiency in organoids that were undetectable in monolayers [123]. Phan et al*.* identified personalized responses of four PDOs which were established from three HGSOCs and one ovarian carcinosarcoma to 240 kinds of kinase inhibitors using automated screening platform within one week from harvesting the original tumor [124]. It was indicated that high-throughput drug screening in PDOs could be feasible, and the timeline of functional testing was compatible with therapeutic decision-making [124].

Sequencing studies have shown that EOCs display extensive inter- and intra-tumor heterogeneity on a genetic level, and functional assays in organoids can be used to address the effects of genomic tumor heterogeneity on therapeutic response. de Witte et al. established a living biobank including 36 whole-genome-characterized PDOs from 23 EOC patients with known clinical histories [114]. At first, seven PDOs (derived from five patients) were exposed to carboplatin and paclitaxel combination treatment in vitro. As a result, significant correlations between organoid sensitivity patterns and clinical outcomes are clearly observed. Then, they investigated the response of all PDOs to a broader range of drugs and drug combinations including PARPi. Comparing IC_50_ with a proposed cut-off based on in vivo plasma PARPi concentrations, all PDOs were found to be resistant to olaparib, rucaparib and niraparib, in line with HRD classification based on whole-genome sequencing (WGS) data. Moreover, for individual patients, PDOs derived from distinct cancer lesions at a single time point exhibited a differential drug response to at least one drug, which represented intra-patient drug response heterogeneity [114]. Nanki et al. utilized a short-term cultured PDO platform for drug sensitivity testing with 23 FDA-approved drugs [115]. A HGSOC model harboring a pathogenic *BRCA*1 variant was found sensitive to DNA damage related agents like cisplatin and olaparib, and the patient’s disease-free period after platinum therapy was longer compared with another HGSOC patient, who showed low response in PDO drug testing [115]. However, genome sequencing showed loss of *BRCA2* in one organoid, but the result of in vitro drug testing showed resistance towards olaparib [123]. These data indicated that responses of PDOs to DNA damage related agents including PARPi often reflect genetic heterogeneity and clinical outcomes. Generating and testing PDOs of multiple tumor locations will provide insights in differential drug responses as a result of tumor heterogeneity. Large scale parallel study of organoids, with comparison to systematically monitored clinical response, will be required to prove the utility of this model system in the clinic.

The available data also demonstrated that patient-derived organoids are a useful model system for rapid assessment of DNA repair defects in ovarian cancer, and highlight their many advantages [108, 110]. Researchers evaluated the HRR capacity of the organoid cultures utilizing functional assays, in particular, assessing the ability to assemble RAD51 protein pre- and post-irradiation at the site of DSBs. Proteins γH2AX to mark DNA damage and geminin to mark cells in S phase were also co-stained. Kopper et al. showed that organoids with a low percentage of geminin positive cells with RAD51 foci were more sensitive to niraparib compared with strong positive group, verifying the correlation between functional HRD and PARPi sensitivity present in EOC organoid [108]. What is noteworthy, Hill et al. suggested the most striking benefit is the clarification of genomic results by the functional assays [110]. In a total of 34 organoid cultures, only two of them (6%) were olaparib sensitive and lacking RAD51 foci. Although HRR pathway gene mutational signature was detected and quantified in five tumors and organoids (2 *gBRCA1*mut, two *gBRCA2*mut, 1 *sRAD51C*mut), which were assumed to be sensitive to PARPi theoretically, they are olaparib resistant and competent for forming RAD51 foci, also consistent with the clinical response. Thus, the magnitude of the HRD signature reflects only the history but not current status of a functional defect in a tumor.

Organoids can be a useful tool in revealing potential PARPi resistant mechanism and choosing rational mono or combined therapy strategies. For example, through drug testing and functional assays, an organoid from a *BRCA1* mutation carrier with acquired PARPi resistance history, exhibited RAD51 foci indicating HRR restoration, but showed sensitivity to carboplatin, prexasertib (Chk1 inhibitor), VE-822 (ATR inhibitor), and gemcitabine. Interestingly, the patient was later treated with prexasertib and exhibited stable disease [110]. For another case harboring a germline *BRCA2* mutation, with de novo pan-resistance to DNA damage agents including olaparib, carboplatin and prexasertib, therapeutic testing may yield useful information even when the drug resistance mechanism is incompletely understood [110]. Furthermore, according to data reported by Hill et al., it was suggested that, since diverse mechanisms involved in protection of replication are not necessarily linked to HRR, combining prexasertib with carboplatin or gemcitabine can enhance replication stress even in fork stable tumors [110]. Thus, it was hypothesized that proper DNA damage repair drug combinations can be effective in tumors without underlying DNA repair defects, which could be tested rapidly in short-term organoids. Beyond gynecological malignancies, PDOs from a subset of solid tumors including endometrial, pancreatic, gastric and metastatic colorectal cancers exhibited differential sensitivity to PARPi despite lacking HRD related genetic alterations [125–127]. These observations provide a strong rationale to explore gene alterations of unknown significance using functional assay-based basket trials and to assess PARPi (either alone or in combination) as an optional treatment.

Furthermore, complementary PDO drug screening and genomic analysis allow linkage of genotypes with drug responsiveness patterns to identify candidate biomarkers for drug response. Recently, Kong J et al. presented a machine-learning framework to identify robust drug biomarkers by taking advantage of network-based analyses using pharmacogenomic data derived from 3D organoid culture models [128]. The biomarkers identified by this approach accurately predicted the drug responses of 114 colorectal cancer patients treated with 5-fluorouracil and 77 bladder cancer patients treated with cisplatin [128]. Establishment of a larger collection of EOC PDOs will provide an opportunity to determine comprehensive, clinically useful genotype-phenotype correlations. With a large collection of drug response data, treatment can potentially be stratified based on genomic or transcriptomics features of specific PDO subtypes, which could, in the future, make organoid derivation dispensable. Moreover, proteomics allows characterization of the proteins and post-translational modifications (PTMs), providing an additional functional layer of information on kinase activity, the dynamic regulation of protein interactions, and cellular signaling networks [129–131]. Data sets of high throughput proteomics could be utilized for training and validation of artificial intelligence (AI) models to address clinical needs. For example, proteomic data from 130 ovarian cancer tissues have been employed to predict platinum drug response using supervised machine learning methods [132, 133]. With regards to PARPi, the incorporation of proteomics into ovarian cancer research might better characterize PDO subtypes to determine therapeutic stratification biomarkers or deciphering underlying resistant mechanisms.

Altogether, a combination of PDO functional or therapeutic assays with genetic features may provide the best predictive accuracy, since genetic features reflect DNA repair ability in the whole cell life cycle while functional assays present this at the time point. The combination could not only predict PARPi sensitivity better, but also further explore the potential resistance mechanism, so as to indirectly or directly guide the choice of individual initial treatment or the combination treatment strategy.

### Challenges and opportunities

**Fig. 3:**
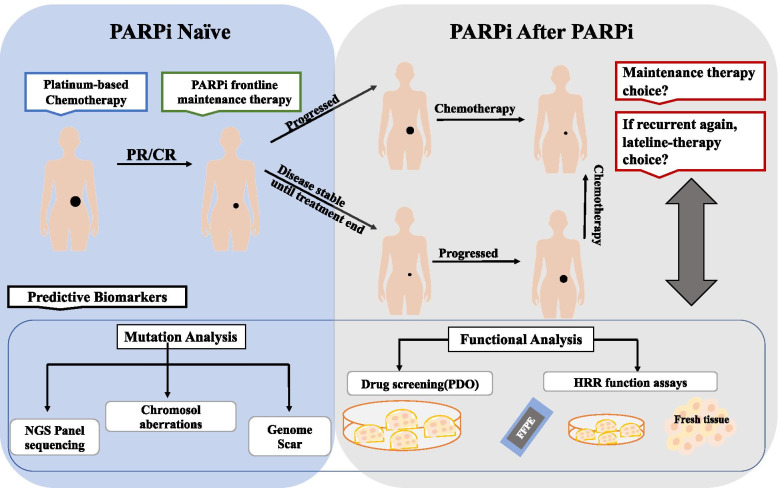
Predictive biomarkers for different settings: PARPi naïve patients & PARPi reused patients

The successful development of PARPi over the last decade constitutes one of the prime examples of success in precision medicine to date, which provides an effective therapeutic option for a subset of ovarian cancers with expansion to other biomarker-driven indications expected in the near future. This article has highlighted the limitations of genomic profiling in predicting response to PARPi especially in PARPi reuse setting and emphasized that functional assays dissecting the specific DNA damage repair defects in a tumor substantially improve the selection of candidates for PARPi. Ovarian cancer PDOs have been established as a promising tumor model that allows for both rapid functional testing and prediction of therapeutic sensitivity with such advantages as high success rate of establishing, variety of tissue sources, short time cost and faithful hereditary preservation. Notably, a combination of functional testing of organoids and genomic analysis allows for the identification of targetable DNA damage repair defects, and also encourages rational combinatorial approaches for overcoming de novo or acquired resistance to PARPi (Fig. 3). However, PDOs do have some limitations that need to be concerned.

First, it should be noted that there is the potential to grow out normal cells instead of transformed cells. Tirac H *et. al* showed that 22% of PDOs from pancreatic patient samples were of normal origins [134], which may be due to insufficient quality of biopsy or insufficient selection of culture condition. Therefore, genomic analysis may be required to confirm tumor origin. Besides, biopsy techniques and culture conditions require optimization and standardization to avoid fibroblast overgrowth or wild-type contamination.

Another important point to consider is the impact of the PDO microenvironment lacking a lasting immune and stroma component on the tumor response to relevant treatment, such as angiogenic and immune checkpoints inhibitors (ICI). For instance, PARPi could increase tumor mutational burden, activate immunosuppressive pathways and reprogram immune microenvironment, offering a novel therapeutic strategy to combine ICI with PARPi [135]. Co-cultures of autologous tumor organoids and peripheral blood lymphocytes might be a platform to induce and analyze tumor-specific T cell responses to epithelial cancers in a personalized manner [136]. Nevertheless, mimicking the whole microenvironment characterizing the tumor is challenging, especially with respect to vascularization of the PDOs.

Finally, an important factor supporting the clinical applicability of organoids is efficiency and reproducibility. Standardizing guidelines for the procedures of tissue manipulation and culture conditions might promote the widespread usage of this technology in clinical settings. In addition, successful establishment of PDOs requires fragments of sufficient size and concentration of proliferating cancer cells. The possibility of having tissue fragments from laparoscopic biopsies and obtaining tumor specimens in the form of MCSs from malignant effusion fluids or circulating tumor cells would allow for higher efficiency of PDO formation. However, median costs for PDO generation and maintenance are relatively high at the moment, thus representing a potential limitation for their application to routine management of patients.

Although longitudinal solid biopsies at diagnosis and relapse can present a significant challenge in ovarian cancers, they remain essential to perform functional assays and to reveal primary or secondary resistance. Future studies with larger patient numbers and longer outcome tracking are required to determine whether organoid testing is a reliable in vitro assay for predicting sensitivity and resistance to PARPi in the clinic.

## Data Availability

Not applicable.

## Abbreviations

EOC: Epithelial ovarian cancer
PARP: Poly ADP-ribose polymerase
HRD: Homologous recombination deficiency
PDO: Patient-derived organoid
SSB: Single strand breaks
DSB: Double-strand breaks
NHEJ: Non-Homologous End Joining
HRR: Homologous recombination repair
NGS: Next-generation sequencing
TCGA: Tumor Cancer Genome Atlas
HGSOC: High-grade serous ovarian cancer
WES: Whole-exome sequencing
WGS: Whole- genome sequencing
LOH: Loss of heterozygosity
TAI: Telomeric allelic imbalance
LST: Large-scale transitions
PFS: Progressed free survival
ORF: Open reading frame
CDK: Cyclin-dependent kinase
PDX: Patient-derived xenograft
MSI: Microsatellite instability
IHC: Immunochemistry
IF: Immunofluorescence
FFPE: Formalin-Fixed and Paraffin-Embedded
3D: Three-dimensional
BRCA: Breast cancer susceptibility gene
ATM: Ataxia telangiectasia mutated
ATR: Ataxia telangiectasia and Rad3 related
FANCF: Fanconi anemia complementation group F
RIF1: Rap1-interacting factor 1
CHD4: Chromodomain Helicase DNA Binding Protein 4
SMARCAL1: SWI/SNF Related, Matrix Associated, Actin Dependent Regulator Of Chromatin, Subfamily A Like 1
EZH2: Enhancer Of Zeste 2 Polycomb Repressive Complex 2 Subunit
PTIP: PAX transcription activation domain interacting protein
c-Met: Hepatocyte growth factor
ABCB1: ATP Binding Cassette Subfamily B
PTM: Post-translational modifications
ICI: Immune checkpoints inhibitors
MCS: Multicellular spheroids

## References

[1] Siegel RL, Miller KD, Jemal A, Statistics C. CA Cancer J Clin. 2017:2017. 10.3322/caac.21387.

[2] Webb PM, Jordan SJ. Epidemiology of epithelial ovarian cancer. Best Pract Res Clin Obstet Gynaecol. 2017. 10.1016/j.bpobgyn.2016.08.006. 10.1016/j.bpobgyn.2016.08.006 27743768

[3] Pignata S, CCS, Du Bois A, et al. Treatment of recurrent ovarian cancer. Ann Oncol. 2017. 10.1093/annonc/mdx441. 10.1093/annonc/mdx441 29232464

[4] Narod S. Can advanced-stage ovarian cancer be cured? Nat Rev Clin Oncol. 2016. 10.1038/nrclinonc.2015.224. 10.1038/nrclinonc.2015.224 26787282

[5] Lord CJ, Ashworth A. PARP inhibitors: Synthetic lethality in the clinic. Science. 2017. 10.1126/science.aam7344. 10.1126/science.aam7344 PMC6175050 28302823

[6] Ledermann JA, Drew Y, Kristeleit RS. Homologous recombination deficiency and ovarian cancer. European Journal of Cancer. 2016. 10.1016/j.ejca.2016.03.005. 10.1016/j.ejca.2016.03.005 27065456

[7] Murai J, Huang SY, Das BB, et al. Trapping of PARP1 and PARP2 by clinical PARP inhibitors. Cancer Res. 2012. 10.1158/0008-5472.Can-12-2753. 10.1158/0008-5472.CAN-12-2753 PMC3528345 23118055

[8] Integrated genomic analyses of ovarian carcinoma. Nature. 2011; 10.1038/nature10166. 10.1038/nature10166 PMC3163504 21720365

[9] Konstantinopoulos PA, Ceccaldi R, Shapiro GI, et al. Homologous Recombination Deficiency: Exploiting the Fundamental Vulnerability of Ovarian Cancer. Cancer Discov. 2015. 10.1158/2159-8290.Cd-15-0714. 10.1158/2159-8290.CD-15-0714 PMC4631624 26463832

[10] Fong PC, Boss DS, Yap TA, et al. Inhibition of poly(ADP-ribose) polymerase in tumors from BRCA mutation carriers. N Engl J Med. 2009. 10.1056/NEJMoa0900212. 10.1056/NEJMoa0900212 19553641

[11] Audeh MW, Carmichael J, Penson RT, et al. Oral poly(ADP-ribose) polymerase inhibitor olaparib in patients with BRCA1 or BRCA2 mutations and recurrent ovarian cancer: a proof-of-concept trial. Lancet. 2010. 10.1016/s0140-6736(10)60893-8. 10.1016/S0140-6736(10)60893-8 20609468

[12] Gelmon KA, Tischkowitz M, Mackay H, et al. Olaparib in patients with recurrent high-grade serous or poorly differentiated ovarian carcinoma or triple-negative breast cancer: a phase 2, multicentre, open-label, non-randomised study. Lancet Oncol. 2011. 10.1016/s1470-2045(11)70214-5. 10.1016/S1470-2045(11)70214-5 21862407

[13] Radhakrishnan SK, Jette N, Lees-Miller SP. Non-homologous end joining: Emerging themes and unanswered questions. DNA Repair. 2014. 10.1016/j.dnarep.2014.01.009. 10.1016/j.dnarep.2014.01.009 PMC4084493 24582502

[14] Ledermann J, Harter P, Gourley C, et al. Olaparib maintenance therapy in patients with platinum-sensitive relapsed serous ovarian cancer: a preplanned retrospective analysis of outcomes by BRCA status in a randomised phase 2 trial. Lancet Oncol. 2014. 10.1016/s1470-2045(14)70228-1. 10.1016/S1470-2045(14)70228-1 24882434

[15] Mirza MR, Monk BJ, Herrstedt J, et al. Niraparib maintenance therapy in platinum-sensitive, recurrent ovarian Cancer. N Engl J Med. 2016. 10.1056/NEJMoa1611310. 10.1056/NEJMoa1611310 27717299

[16] Coleman RL, Oza AM, Lorusso D, et al. Rucaparib maintenance treatment for recurrent ovarian carcinoma after response to platinum therapy (ARIEL3): a randomised, double-blind, placebo-controlled, phase 3 trial. Lancet. 2017. 10.1016/s0140-6736(17)32440-6. 10.1016/S0140-6736(17)32440-6 PMC5901715 28916367

[17] Moore K, Colombo N, Scambia G, et al. Maintenance Olaparib in Patients with Newly Diagnosed Advanced Ovarian Cancer. N Engl J Med. 2018. 10.1056/NEJMoa1810858. 10.1056/NEJMoa1810858 30345884

[18] Li H, Liu ZY, Wu N, et al. PARP inhibitor resistance: the underlying mechanisms and clinical implications. Mol Cancer. 2020. 10.1186/s12943-020-01227-0. 10.1186/s12943-020-01227-0 PMC7305609 32563252

[19] Dutta D, Heo I, Clevers H. Disease modeling in stem cell-derived 3D organoid systems. Trends Mol Med. 2017. 10.1016/j.molmed.2017.02.007. 10.1016/j.molmed.2017.02.007 28341301

[20] Drost J, Clevers H. Organoids in cancer research. Nat Rev Cancer. 2018. 10.1038/s41568-018-0007-6. 10.1038/s41568-018-0007-6 29692415

[21] Bleijs M, van de Wetering M, Clevers H, et al. Xenograft and organoid model systems in cancer research. Embo J. 2019. 10.15252/embj.2019101654. 10.15252/embj.2019101654 PMC6670015 31282586

[22] Mouw KW, Goldberg MS, Konstantinopoulos PA, et al. DNA Damage and Repair Biomarkers of Immunotherapy Response. Cancer Discov. 2017. 10.1158/2159-8290.Cd-17-0226. 10.1158/2159-8290.CD-17-0226 PMC5659200 28630051

[23] Brown JS, O'Carrigan B, Jackson SP, et al. Targeting DNA Repair in Cancer: Beyond PARP Inhibitors. Cancer Discov. 2017. 10.1158/2159-8290.Cd-16-0860. 10.1158/2159-8290.CD-16-0860 PMC5300099 28003236

[24] Plummer R. Perspective on the pipeline of drugs being developed with modulation of DNA damage as a target. Clin Cancer Res. 2010. 10.1158/1078-0432.Ccr-10-0984. 10.1158/1078-0432.CCR-10-0984 20823148

[25] Forment JV, O'Connor MJ. Targeting the replication stress response in cancer. Pharmacol Ther. 2018. 10.1016/j.pharmthera.2018.03.005. 10.1016/j.pharmthera.2018.03.005 29580942

[26] Bryant HE, Petermann E, Schultz N, et al. PARP is activated at stalled forks to mediate Mre11-dependent replication restart and recombination. EMBO J. 2009. 10.1038/emboj.2009.206. 10.1038/emboj.2009.206 PMC2738702 19629035

[27] De Vos M, Schreiber V, Dantzer F. The diverse roles and clinical relevance of PARPs in DNA damage repair: current state of the art. Biochem Pharmacol. 2012. 10.1016/j.bcp.2012.03.018. 10.1016/j.bcp.2012.03.018 22469522

[28] Eustermann S, Wu WF, Langelier MF, et al. Structural basis of detection and signaling of DNA single-Strand breaks by human PARP-1. Mol Cell. 2015. 10.1016/j.molcel.2015.10.032. 10.1016/j.molcel.2015.10.032 PMC4678113 26626479

[29] Krishnakumar R, Kraus WL. The PARP side of the nucleus: molecular actions, physiological outcomes, and clinical targets. Mol Cell. 2010. 10.1016/j.molcel.2010.06.017. 10.1016/j.molcel.2010.06.017 PMC2923840 20603072

[30] Wei H, Yu X. Functions of PARylation in DNA Damage Repair Pathways. Genomics Proteomics Bioinformatics. 2016. 10.1016/j.gpb.2016.05.001. 10.1016/j.gpb.2016.05.001 PMC4936651 27240471

[31] Moynahan ME, Jasin M. Mitotic homologous recombination maintains genomic stability and suppresses tumorigenesis. Nat Rev Mol Cell Biol. 2010. 10.1038/nrm2851. 10.1038/nrm2851 PMC3261768 20177395

[32] Radhakrishnan SK, Jette N, Lees-Miller SP. Non-homologous end joining: emerging themes and unanswered questions. DNA Repair (Amst). 2014. 10.1016/j.dnarep.2014.01.009. 10.1016/j.dnarep.2014.01.009 PMC4084493 24582502

[33] Aymard F, Bugler B, Schmidt CK, et al. Transcriptionally active chromatin recruits homologous recombination at DNA double-strand breaks. Nat Struct Mol Biol. 2014. 10.1038/nsmb.2796. 10.1038/nsmb.2796 PMC4300393 24658350

[34] Farmer H, McCabe N, Lord CJ, et al. Targeting the DNA repair defect in BRCA mutant cells as a therapeutic strategy. Nature. 2005. 10.1038/nature03445. 10.1038/nature03445 15829967

[35] Bryant HE, Schultz N, Thomas HD, et al. Specific killing of BRCA2-deficient tumours with inhibitors of poly(ADP-ribose) polymerase. Nature. 2005. 10.1038/nature03443. 10.1038/nature03443 15829966

[36] McCabe N, Turner NC, Lord CJ, et al. Deficiency in the repair of DNA damage by homologous recombination and sensitivity to poly(ADP-ribose) polymerase inhibition. Cancer Res. 2006. 10.1158/0008-5472.Can-06-0140. 10.1158/0008-5472.CAN-06-0140 16912188

[37] Bajrami I, Frankum JR, Konde A, et al. Genome-wide profiling of genetic synthetic lethality identifies CDK12 as a novel determinant of PARP1/2 inhibitor sensitivity. Cancer Res. 2014. 10.1158/0008-5472.Can-13-2541. 10.1158/0008-5472.CAN-13-2541 PMC4886090 24240700

[38] Murai J, Huang SY, Renaud A, et al. Stereospecific PARP trapping by BMN 673 and comparison with olaparib and rucaparib. Mol Cancer Ther. 2014. 10.1158/1535-7163.Mct-13-0803. 10.1158/1535-7163.MCT-13-0803 PMC3946062 24356813

[39] Mittica G, Ghisoni E, Giannone G, et al. PARP Inhibitors in Ovarian Cancer. Recent Pat Anticancer Drug Discov. 2018. 10.2174/1574892813666180305165256. 10.2174/1574892813666180305165256 29512470

[40] Fong PC, Yap TA, Boss DS, et al. Poly(ADP)-ribose polymerase inhibition: frequent durable responses in BRCA carrier ovarian cancer correlating with platinum-free interval. J Clin Oncol. 2010. 10.1200/jco.2009.26.9589. 10.1200/JCO.2009.26.9589 20406929

[41] Pennington KP, Walsh T, Harrell MI, et al. Germline and somatic mutations in homologous recombination genes predict platinum response and survival in ovarian, fallopian tube, and peritoneal carcinomas. Clin Cancer Res. 2014. 10.1158/1078-0432.Ccr-13-2287. 10.1158/1078-0432.CCR-13-2287 PMC3944197 24240112

[42] Norquist BM, Brady MF, Harrell MI, et al. Mutations in homologous recombination genes and outcomes in ovarian carcinoma patients in GOG 218: an NRG oncology/gynecologic oncology group study. Clin Cancer Res. 2018. 10.1158/1078-0432.Ccr-17-1327. 10.1158/1078-0432.CCR-17-1327 PMC5815909 29191972

[43] Hodgson DR, Dougherty BA, Lai Z, et al. Candidate biomarkers of PARP inhibitor sensitivity in ovarian cancer beyond the BRCA genes. Br J Cancer. 2018. 10.1038/s41416-018-0274-8. 10.1038/s41416-018-0274-8 PMC6265286 30353044

[44] Swisher EM, Lin KK, Oza AM, et al. Rucaparib in relapsed, platinum-sensitive high-grade ovarian carcinoma (ARIEL2 part 1): an international, multicentre, open-label, phase 2 trial. Lancet Oncol. 2017. 10.1016/s1470-2045(16)30559-9. 10.1016/S1470-2045(16)30559-9 27908594

[45] Mateo J, Porta N, Bianchini D, et al. Olaparib in patients with metastatic castration-resistant prostate cancer with DNA repair gene aberrations (TOPARP-B): a multicentre, open-label, randomised, phase 2 trial. Lancet Oncol. 2020. 10.1016/s1470-2045(19)30684-9. 10.1016/S1470-2045(19)30684-9 PMC6941219 31806540

[46] Li H, LaDuca H, Pesaran T, et al. Classification of variants of uncertain significance in BRCA1 and BRCA2 using personal and family history of cancer from individuals in a large hereditary cancer multigene panel testing cohort. Genet Med. 2020. 10.1038/s41436-019-0729-1. 10.1038/s41436-019-0729-1 PMC7118020 31853058

[47] Cartegni L, Chew SL, Krainer AR. Listening to silence and understanding nonsense: exonic mutations that affect splicing. Nat Rev Genet. 2002. 10.1038/nrg775. 10.1038/nrg775 11967553

[48] Bonnet C, Krieger S, Vezain M, et al. Screening BRCA1 and BRCA2 unclassified variants for splicing mutations using reverse transcription PCR on patient RNA and an ex vivo assay based on a splicing reporter minigene. J Med Genet. 2008. 10.1136/jmg.2007.056895. 10.1136/jmg.2007.056895 18424508

[49] Alexandrov LB, Kim J, Haradhvala NJ, et al. The repertoire of mutational signatures in human cancer. Nature. 2020. 10.1038/s41586-020-1943-3. 10.1038/s41586-020-1943-3 PMC7054213 32025018

[50] Watkins JA, Irshad S, Grigoriadis A, et al. Genomic scars as biomarkers of homologous recombination deficiency and drug response in breast and ovarian cancers. Breast Cancer Res. 2014. 10.1186/bcr3670. 10.1186/bcr3670 PMC4053155 25093514

[51] Polak P, Kim J, Braunstein LZ, et al. A mutational signature reveals alterations underlying deficient homologous recombination repair in breast cancer. Nat Genet. 2017. 10.1038/ng.3934. 10.1038/ng.3934 PMC7376751 28825726

[52] Abkevich V, Timms KM, Hennessy BT, et al. Patterns of genomic loss of heterozygosity predict homologous recombination repair defects in epithelial ovarian cancer. Br J Cancer. 2012. 10.1038/bjc.2012.451. 10.1038/bjc.2012.451 PMC3493866 23047548

[53] Talens F, Jalving M, Gietema JA, et al. Therapeutic targeting and patient selection for cancers with homologous recombination defects. Expert Opin Drug Discov. 2017. 10.1080/17460441.2017.1322061. 10.1080/17460441.2017.1322061 28425306

[54] Davies H, Glodzik D, Morganella S, et al. HRDetect is a predictor of BRCA1 and BRCA2 deficiency based on mutational signatures. Nat Med. 2017. 10.1038/nm.4292. 10.1038/nm.4292 PMC5833945 28288110

[55] Ceccaldi R, Sarangi P, D'Andrea AD. The Fanconi anaemia pathway: new players and new functions. Nat Rev Mol Cell Biol. 2016. 10.1038/nrm.2016.48. 10.1038/nrm.2016.48 27145721

[56] Graeser M, McCarthy A, Lord CJ, et al. A marker of homologous recombination predicts pathologic complete response to neoadjuvant chemotherapy in primary breast cancer. Clin Cancer Res. 2010. 10.1158/1078-0432.Ccr-10-1027. 10.1158/1078-0432.CCR-10-1027 PMC3432445 20802015

[57] Asakawa H, Koizumi H, Koike A, et al. Prediction of breast cancer sensitivity to neoadjuvant chemotherapy based on status of DNA damage repair proteins. Breast Cancer Res. 2010. 10.1186/bcr2486. 10.1186/bcr2486 PMC2879561 20205718

[58] Kubelac P, Genestie C, Auguste A, et al. Changes in DNA damage response markers with treatment in advanced ovarian Cancer. Cancers (Basel). 2020. 10.3390/cancers12030707. 10.3390/cancers12030707 PMC7140046 32192091

[59] Willers H, Taghian AG, Luo CM, et al. Utility of DNA repair protein foci for the detection of putative BRCA1 pathway defects in breast cancer biopsies. Mol Cancer Res. 2009. 10.1158/1541-7786.Mcr-09-0149. 10.1158/1541-7786.MCR-09-0149 PMC4239295 19671671

[60] Naipal KA, Verkaik NS, Ameziane N, et al. Functional ex vivo assay to select homologous recombination-deficient breast tumors for PARP inhibitor treatment. Clin Cancer Res. 2014. 10.1158/1078-0432.Ccr-14-0571. 10.1158/1078-0432.CCR-14-0571 24963051

[61] Meijer TG, Verkaik NS, Sieuwerts AM, et al. Functional Ex Vivo Assay Reveals Homologous Recombination Deficiency in Breast Cancer Beyond BRCA Gene Defects. Clin Cancer Res. 2018. 10.1158/1078-0432.Ccr-18-0063. 10.1158/1078-0432.CCR-18-0063 30139880

[62] van Wijk LM, Vermeulen S, Meijers M, et al. The RECAP test rapidly and reliably identifies homologous recombination-deficient ovarian carcinomas. Cancers (Basel). 2020. 10.3390/cancers12102805. 10.3390/cancers12102805 PMC7650677 33003546

[63] Cruz C, Castroviejo-Bermejo M, Gutiérrez-Enríquez S, et al. RAD51 foci as a functional biomarker of homologous recombination repair and PARP inhibitor resistance in germline BRCA-mutated breast cancer. Ann Oncol. 2018. 10.1093/annonc/mdy099. 10.1093/annonc/mdy099 PMC5961353 29635390

[64] Castroviejo-Bermejo M, Cruz C, Llop-Guevara A, et al. A RAD51 assay feasible in routine tumor samples calls PARP inhibitor response beyond BRCA mutation. EMBO Mol Med. 2018. 10.15252/emmm.201809172. 10.15252/emmm.201809172 PMC6284440 30377213

[65] Färkkilä A, Gulhan DC, Casado J, et al. Immunogenomic profiling determines responses to combined PARP and PD-1 inhibition in ovarian cancer. Nat Commun. 2020. 10.1038/s41467-020-15315-8. 10.1038/s41467-020-15315-8 PMC7081234 32193378

[66] Edwards SL, Brough R, Lord CJ, et al. Resistance to therapy caused by intragenic deletion in BRCA2. Nature. 2008. 10.1038/nature06548. 10.1038/nature06548 18264088

[67] Barber LJ, Sandhu S, Chen L, et al. Secondary mutations in BRCA2 associated with clinical resistance to a PARP inhibitor. J Pathol. 2013. 10.1002/path.4140. 10.1002/path.4140 23165508

[68] Sakai W, Swisher EM, Karlan BY, et al. Secondary mutations as a mechanism of cisplatin resistance in BRCA2-mutated cancers. Nature. 2008. 10.1038/nature06633. 10.1038/nature06633 PMC2577037 18264087

[69] Lord CJ, Ashworth A. BRCAness revisited. Nat Rev Cancer. 2016. 10.1038/nrc.2015.21. 10.1038/nrc.2015.21 26775620

[70] Lin KK, Harrell MI, Oza AM, et al. BRCA Reversion Mutations in Circulating Tumor DNA Predict Primary and Acquired Resistance to the PARP Inhibitor Rucaparib in High-Grade Ovarian Carcinoma. Cancer Discov. 2019. 10.1158/2159-8290.Cd-18-0715. 10.1158/2159-8290.CD-18-0715 30425037

[71] Symington LS, Gautier J. Double-strand break end resection and repair pathway choice. Annu Rev Genet. 2011. 10.1146/annurev-genet-110410-132435. 10.1146/annurev-genet-110410-132435 21910633

[72] Di Virgilio M, Callen E, Yamane A, et al. Rif1 prevents resection of DNA breaks and promotes immunoglobulin class switching. Science. 2013. 10.1126/science.1230624. 10.1126/science.1230624 PMC3815530 23306439

[73] Zimmermann M, Lottersberger F, Buonomo SB, et al. 53BP1 regulates DSB repair using Rif1 to control 5′ end resection. Science. 2013. 10.1126/science.1231573. 10.1126/science.1231573 PMC3664841 23306437

[74] Noordermeer SM, Adam S, Setiaputra D, et al. The shieldin complex mediates 53BP1-dependent DNA repair. Nature. 2018. 10.1038/s41586-018-0340-7. 10.1038/s41586-018-0340-7 PMC6141009 30022168

[75] Ghezraoui H, Oliveira C, Becker JR, et al. 53BP1 cooperation with the REV7-shieldin complex underpins DNA structure-specific NHEJ. Nature. 2018. 10.1038/s41586-018-0362-1. 10.1038/s41586-018-0362-1 PMC6989217 30046110

[76] Jaspers JE, Kersbergen A, Boon U, et al. Loss of 53BP1 causes PARP inhibitor resistance in Brca1-mutated mouse mammary tumors. Cancer Discov. 2013. 10.1158/2159-8290.Cd-12-0049. 10.1158/2159-8290.CD-12-0049 PMC7518105 23103855

[77] Xu G, Chapman JR, Brandsma I, et al. REV7 counteracts DNA double-strand break resection and affects PARP inhibition. Nature. 2015. 10.1038/nature14328. 10.1038/nature14328 PMC4671316 25799992

[78] Gupta R, Somyajit K, Narita T, et al. DNA Repair Network Analysis Reveals Shieldin as a Key Regulator of NHEJ and PARP Inhibitor Sensitivity. Cell. 2018. 10.1016/j.cell.2018.03.050. 10.1016/j.cell.2018.03.050 PMC8108093 29656893

[79] Hustedt N, Durocher D. The control of DNA repair by the cell cycle. Nat Cell Biol. 2016. 10.1038/ncb3452. 10.1038/ncb3452 28008184

[80] Berti M, Ray Chaudhuri A, Thangavel S, et al. Human RECQ1 promotes restart of replication forks reversed by DNA topoisomerase I inhibition. Nat Struct Mol Biol. 2013. 10.1038/nsmb.2501. 10.1038/nsmb.2501 PMC3897332 23396353

[81] Schlacher K, Christ N, Siaud N, et al. Double-strand break repair-independent role for BRCA2 in blocking stalled replication fork degradation by MRE11. Cell. 2011. 10.1016/j.cell.2011.03.041. 10.1016/j.cell.2011.03.041 PMC3261725 21565612

[82] Schlacher K, Wu H, Jasin M. A distinct replication fork protection pathway connects Fanconi anemia tumor suppressors to RAD51-BRCA1/2. Cancer Cell. 2012. 10.1016/j.ccr.2012.05.015. 10.1016/j.ccr.2012.05.015 PMC3954744 22789542

[83] Liao H, Ji F, Helleday T, et al. Mechanisms for stalled replication fork stabilization: new targets for synthetic lethality strategies in cancer treatments. EMBO Rep. 2018. 10.15252/embr.201846263. 10.15252/embr.201846263 PMC6123652 30108055

[84] Ray Chaudhuri A, Callen E, Ding X, et al. Replication fork stability confers chemoresistance in BRCA-deficient cells. Nature. 2016. 10.1038/nature18325. 10.1038/nature18325 PMC4959813 27443740

[85] Ying S, Hamdy FC, Helleday T. Mre11-dependent degradation of stalled DNA replication forks is prevented by BRCA2 and PARP1. Cancer Res. 2012. 10.1158/0008-5472.Can-11-3417. 10.1158/0008-5472.CAN-11-3417 22447567

[86] Polato F, Callen E, Wong N, et al. CtIP-mediated resection is essential for viability and can operate independently of BRCA1. J Exp Med. 2014. 10.1084/jem.20131939. 10.1084/jem.20131939 PMC4042650 24842372

[87] Guillemette S, Serra RW, Peng M, et al. Resistance to therapy in BRCA2 mutant cells due to loss of the nucleosome remodeling factor CHD4. Genes Dev. 2015. 10.1101/gad.256214.114. 10.1101/gad.256214.114 PMC4358401 25737278

[88] Kolinjivadi AM, Sannino V, De Antoni A, et al. Smarcal1-mediated fork reversal triggers Mre11-dependent degradation of nascent DNA in the absence of Brca2 and stable Rad51 nucleofilaments. Mol Cell. 2017. 10.1016/j.molcel.2017.07.001. 10.1016/j.molcel.2017.07.001 PMC5594205 28757209

[89] Lok BH, Gardner EE, Schneeberger VE, et al. PARP inhibitor activity correlates with SLFN11 expression and demonstrates synergy with Temozolomide in small cell lung Cancer. Clin Cancer Res. 2017. 10.1158/1078-0432.Ccr-16-1040. 10.1158/1078-0432.CCR-16-1040 PMC5241177 27440269

[90] Rondinelli B, Gogola E, Yücel H, et al. EZH2 promotes degradation of stalled replication forks by recruiting MUS81 through histone H3 trimethylation. Nat Cell Biol. 2017. 10.1038/ncb3626. 10.1038/ncb3626 29035360

[91] Ding X, Ray Chaudhuri A, Callen E, et al. Synthetic viability by BRCA2 and PARP1/ARTD1 deficiencies. Nat Commun. 2016. 10.1038/ncomms12425. 10.1038/ncomms12425 PMC4979061 27498558

[92] Kondrashova O, Topp M, Nesic K, et al. Methylation of all BRCA1 copies predicts response to the PARP inhibitor rucaparib in ovarian carcinoma. Nat Commun. 2018. 10.1038/s41467-018-05564-z. 10.1038/s41467-018-05564-z PMC6162272 30266954

[93] Du Y, Yamaguchi H, Wei Y, et al. Blocking c-met-mediated PARP1 phosphorylation enhances anti-tumor effects of PARP inhibitors. Nat Med. 2016. 10.1038/nm.4032. 10.1038/nm.4032 PMC4754671 26779812

[94] Christie EL, Pattnaik S, Beach J, et al. Multiple ABCB1 transcriptional fusions in drug resistant high-grade serous ovarian and breast cancer. Nat Commun. 2019. 10.1038/s41467-019-09312-9. 10.1038/s41467-019-09312-9 PMC6426934 30894541

[95] Ibrahim YH, García-García C, Serra V, et al. PI3K inhibition impairs BRCA1/2 expression and sensitizes BRCA-proficient triple-negative breast cancer to PARP inhibition. Cancer Discov. 2012. 10.1158/2159-8290.Cd-11-0348. 10.1158/2159-8290.CD-11-0348 PMC5125254 22915752

[96] Johnson SF, Cruz C, Greifenberg AK, et al. CDK12 inhibition reverses De novo and acquired PARP inhibitor resistance in BRCA wild-type and mutated models of triple-negative breast Cancer. Cell Rep. 2016. 10.1016/j.celrep.2016.10.077. 10.1016/j.celrep.2016.10.077 PMC5176643 27880910

[97] Liszczak G, Diehl KL, Dann GP, et al. Acetylation blocks DNA damage-induced chromatin ADP-ribosylation. Nat Chem Biol. 2018. 10.1038/s41589-018-0097-1. 10.1038/s41589-018-0097-1 PMC6505472 30013063

[98] Konstantinopoulos PA, Barry WT, Birrer M, et al. Olaparib and α-specific PI3K inhibitor alpelisib for patients with epithelial ovarian cancer: a dose-escalation and dose-expansion phase 1b trial. Lancet Oncol. 2019. 10.1016/s1470-2045(18)30905-7. 10.1016/S1470-2045(18)30905-7 PMC7025391 30880072

[99] Lheureux S, Oaknin A, Garg S, et al. EVOLVE: A Multicenter Open-Label Single-Arm Clinical and Translational Phase II Trial of Cediranib Plus Olaparib for Ovarian Cancer after PARP Inhibition Progression. Clin Cancer Res. 2020. 10.1158/1078-0432.Ccr-19-4121. 10.1158/1078-0432.CCR-19-4121 32444417

[100] Ben-David U, Siranosian B, Ha G, et al. Genetic and transcriptional evolution alters cancer cell line drug response. Nature. 2018. 10.1038/s41586-018-0409-3. 10.1038/s41586-018-0409-3 PMC6522222 30089904

[101] Ben-David U, Ha G, Tseng YY, et al. Patient-derived xenografts undergo mouse-specific tumor evolution. Nat Genet. 2017. 10.1038/ng.3967. 10.1038/ng.3967 PMC5659952 28991255

[102] Rossi G, Manfrin A, Lutolf MP. Progress and potential in organoid research. Nat Rev Genet. 2018. 10.1038/s41576-018-0051-9. 10.1038/s41576-018-0051-9 30228295

[103] Broutier L, Mastrogiovanni G, Verstegen MM, et al. Human primary liver cancer-derived organoid cultures for disease modeling and drug screening. Nat Med. 2017. 10.1038/nm.4438. 10.1038/nm.4438 PMC5722201 29131160

[104] Gao D, Vela I, Sboner A, et al. Organoid cultures derived from patients with advanced prostate cancer. Cell. 2014. 10.1016/j.cell.2014.08.016. 10.1016/j.cell.2014.08.016 PMC4237931 25201530

[105] Sachs N, de Ligt J, Kopper O, et al. A Living Biobank of Breast Cancer Organoids Captures Disease Heterogeneity. Cell. 2018. 10.1016/j.cell.2017.11.010. 10.1016/j.cell.2017.11.010 29224780

[106] van de Wetering M, Francies HE, Francis JM, et al. Prospective derivation of a living organoid biobank of colorectal cancer patients. Cell. 2015. 10.1016/j.cell.2015.03.053. 10.1016/j.cell.2015.03.053 PMC6428276 25957691

[107] Yan HHN, Siu HC, Law S, et al. A Comprehensive Human Gastric Cancer Organoid Biobank Captures Tumor Subtype Heterogeneity and Enables Therapeutic Screening. Cell Stem Cell. 2018. 10.1016/j.stem.2018.09.016. 10.1016/j.stem.2018.09.016 30344100

[108] Kopper O, de Witte CJ, Lõhmussaar K, et al. An organoid platform for ovarian cancer captures intra- and interpatient heterogeneity. Nat Med. 2019. 10.1038/s41591-019-0422-6. 10.1038/s41591-019-0422-6 31011202

[109] Kessler M, Hoffmann K, Brinkmann V, et al. The Notch and Wnt pathways regulate stemness and differentiation in human fallopian tube organoids. Nat Commun. 2015. 10.1038/ncomms9989. 10.1038/ncomms9989 PMC4686873 26643275

[110] Hill SJ, Decker B, Roberts EA, et al. Prediction of DNA Repair Inhibitor Response in Short-Term Patient-Derived Ovarian Cancer. Organoids Cancer Discov. 2018. 10.1158/2159-8290.Cd-18-0474. 10.1158/2159-8290.CD-18-0474 PMC6365285 30213835

[111] Maenhoudt N, Defraye C, Boretto M, et al. Developing Organoids from Ovarian Cancer as Experimental and Preclinical Models. Stem Cell Reports. 2020. 10.1016/j.stemcr.2020.03.004. 10.1016/j.stemcr.2020.03.004 PMC7160357 32243841

[112] Maru Y, Tanaka N, Itami M, et al. Efficient use of patient-derived organoids as a preclinical model for gynecologic tumors. Gynecol Oncol. 2019. 10.1016/j.ygyno.2019.05.005. 10.1016/j.ygyno.2019.05.005 31101504

[113] Chen H, Gotimer K, De Souza C, et al. Short-term organoid culture for drug sensitivity testing of high-grade serous carcinoma. Gynecol Oncol. 2020. 10.1016/j.ygyno.2020.03.026. 10.1016/j.ygyno.2020.03.026 PMC7819712 32253045

[114] de Witte CJ, Espejo Valle-Inclan J, Hami N, et al. Patient-Derived Ovarian Cancer Organoids Mimic Clinical Response and Exhibit Heterogeneous Inter- and Intrapatient Drug Responses. Cell Rep. 2020. 10.1016/j.celrep.2020.107762. 10.1016/j.celrep.2020.107762 32553164

[115] Nanki Y, Chiyoda T, Hirasawa A, et al. Patient-derived ovarian cancer organoids capture the genomic profiles of primary tumours applicable for drug sensitivity and resistance testing. Sci Rep. 2020. 10.1038/s41598-020-69488-9. 10.1038/s41598-020-69488-9 PMC7387538 32724113

[116] Hoffmann K, Berger H, Kulbe H, et al. Stable expansion of high-grade serous ovarian cancer organoids requires a low-Wnt environment. Embo J. 2020. 10.15252/embj.2019104013. 10.15252/embj.2019104013 PMC7073464 32009247

[117] Soragni A, Janzen DM, Johnson LM, et al. A designed inhibitor of p53 aggregation rescues p53 tumor suppression in ovarian carcinomas. Cancer Cell. 2016. 10.1016/j.ccell.2015.12.002. 10.1016/j.ccell.2015.12.002 PMC4733364 26748848

[118] Bi J, Thiel KW, Litman JM, et al. Characterization of a TP53 somatic variant of unknown function from an ovarian Cancer patient using organoid culture and computational modeling. Clin Obstet Gynecol. 2020. 10.1097/grf.0000000000000516. 10.1097/GRF.0000000000000516 PMC7521672 31876640

[119] Florent R, Weiswald LB, Lambert B, et al. Bim, Puma and Noxa upregulation by Naftopidil sensitizes ovarian cancer to the BH3-mimetic ABT-737 and the MEK inhibitor Trametinib. Cell Death Dis. 2020. 10.1038/s41419-020-2588-8. 10.1038/s41419-020-2588-8 PMC7235085 32424251

[120] Sun H, Wang H, Wang X, et al. Aurora-a/SOX8/FOXK1 signaling axis promotes chemoresistance via suppression of cell senescence and induction of glucose metabolism in ovarian cancer organoids and cells. Theranostics. 2020. 10.7150/thno.43811. 10.7150/thno.43811 PMC7295065 32550913

[121] Vernon M, Lambert B, Meryet-Figuière M, et al. Functional miRNA screening identifies wide-ranging antitumor properties of miR-3622b-5p and reveals a new therapeutic combination strategy in ovarian tumor organoids. Mol Cancer Ther. 2020. 10.1158/1535-7163.Mct-19-0510. 10.1158/1535-7163.MCT-19-0510 32371581

[122] Shigeta S, Lui GYL, Shaw R, et al. Targeting BET proteins BRD2 and BRD3 in combination with PI3K-AKT inhibition as a therapeutic strategy for ovarian clear cell carcinoma. Mol Cancer Ther. 2021. 10.1158/1535-7163.Mct-20-0809. 10.1158/1535-7163.MCT-20-0809 PMC8026742 33509905

[123] Jabs J, Zickgraf FM, Park J, et al. Screening drug effects in patient-derived cancer cells links organoid responses to genome alterations. Mol Syst Biol. 2017. 10.15252/msb.20177697. 10.15252/msb.20177697 PMC5731348 29180611

[124] Phan N, Hong JJ, Tofig B, et al. A simple high-throughput approach identifies actionable drug sensitivities in patient-derived tumor organoids. Commun Biol. 2019. 10.1038/s42003-019-0305-x. 10.1038/s42003-019-0305-x PMC6389967 30820473

[125] Driehuis E, van Hoeck A, Moore K, et al. Pancreatic cancer organoids recapitulate disease and allow personalized drug screening. Proceedings of the National Academy of Sciences. 2019. 10.1073/pnas.1911273116. 10.1073/pnas.1911273116 PMC6936689 31818951

[126] Pauli C, Hopkins BD, Prandi D, et al. Personalized In Vitro and In Vivo Cancer Models to Guide Precision Medicine. Cancer Discov. 2017. 10.1158/2159-8290.Cd-16-1154. 10.1158/2159-8290.CD-16-1154 PMC5413423 28331002

[127] Arena S, Corti G, Durinikova E, et al. A Subset of Colorectal Cancers with Cross-Sensitivity to Olaparib and Oxaliplatin. Clin Cancer Res. 2020. 10.1158/1078-0432.Ccr-19-2409. 10.1158/1078-0432.CCR-19-2409 31831554

[128] Kong J, Lee H, Kim D, et al. Network-based machine learning in colorectal and bladder organoid models predicts anti-cancer drug efficacy in patients. Nat Commun. 2020. 10.1038/s41467-020-19313-8. 10.1038/s41467-020-19313-8 PMC7599252 33127883

[129] Zhang H, Liu T, Zhang Z, et al. Integrated Proteogenomic Characterization of Human High-Grade Serous Ovarian. Cancer Cell. 2016. 10.1016/j.cell.2016.05.069. 10.1016/j.cell.2016.05.069 PMC4967013 27372738

[130] Song G, Chen L, Zhang B, et al. Proteome-wide Tyrosine Phosphorylation Analysis Reveals Dysregulated Signaling Pathways in Ovarian Tumors. Mol Cell Proteomics. 2019. 10.1074/mcp.RA118.000851. 10.1074/mcp.RA118.000851 PMC6398206 30523211

[131] McDermott JE, Arshad OA, Petyuk VA, et al. Proteogenomic characterization of ovarian HGSC implicates mitotic kinases, Replication Stress in Observed Chromosomal Instability. Cell Rep Med. 2020. 10.1016/j.xcrm.2020.100004. 10.1016/j.xcrm.2020.100004 PMC7289043 32529193

[132] Yu KH, Levine DA, Zhang H, et al. J Proteome Res. 2016. 10.1021/acs.jproteome.5b01129.

[133] Xiao Q, Zhang F, Xu L, et al. High-throughput proteomics and AI for cancer biomarker discovery. Adv Drug Deliv Rev. 2021. 10.1016/j.addr.2021.113844. 10.1016/j.addr.2021.113844 34182017

[134] Tiriac H, Belleau P, Engle DD, et al. Organoid Profiling Identifies Common Responders to Chemotherapy in Pancreatic. Cancer Cancer Discov. 2018. 10.1158/2159-8290.Cd-18-0349. 10.1158/2159-8290.CD-18-0349 PMC6125219 29853643

[135] Peyraud F, Italiano A. Combined PARP inhibition and immune checkpoint therapy in solid tumors. Cancers (Basel). 2020. 10.3390/cancers12061502. 10.3390/cancers12061502 PMC7352466 32526888

[136] Dijkstra KK, Cattaneo CM, Weeber F, et al. Generation of Tumor-Reactive T Cells by Co-culture of Peripheral Blood Lymphocytes and Tumor Organoids. Cell. 2018. 10.1016/j.cell.2018.07.009. 10.1016/j.cell.2018.07.009 PMC6558289 30100188

